# A new polymorph of 5,5′-(ethane-1,2-di­yl)bis­(1*H*-tetra­zole)

**DOI:** 10.1107/S1600536812008483

**Published:** 2012-03-07

**Authors:** Cheng-Fang Qiao, Chun-Sheng Zhou, Qing Wei, Zheng-Qiang Xia

**Affiliations:** a Department of Chemistry and Chemical Engineering, Shaanxi Key Laboratory of Comprehensive Utilization of Tailing Resources, Shangluo University, Shangluo 726000, Shaanxi, People’s Republic of China; b College of Chemistry and Materials Science, Northwest University, Xi’an 710069, Shaanxi, People’s Republic of China

## Abstract

The asymmetric unit of the title compound, C_4_H_6_N_8_, contains a quarter of the mol­ecule, which possesses a crystallographically imposed centre of symmetry with all non-H atoms situated on a mirror plane. The crystal packing exhibits inter­molecular N—H⋯N hydrogen bonds and π–π stacking inter­actions between the tetra­zole rings of adjacent mol­ecules [centroid–centroid distance = 3.4402 (10) Å].

## Related literature

For the previously reported polymorph, see: Shen *et al.* (2011 ▶). For the synthesis of the title compound and for related structures, see: Chafin *et al.* (2008 ▶); Diop *et al.* (2002 ▶). For the application of tetra­zole derivatives in coordination chemistry and energetic materials, see: Zhao *et al.* (2008 ▶); Singh *et al.* (2006 ▶).
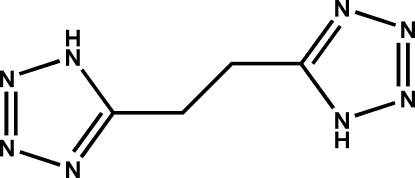


## Experimental

### 

#### Crystal data

C_4_H_6_N_8_
*M* *_r_* = 166.17
Monoclinic, 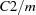

*a* = 10.951 (3) Å
*b* = 6.678 (2) Å
*c* = 5.0329 (14) Å
β = 114.250 (4)°
*V* = 335.58 (17) Å^3^
*Z* = 2
Mo *K*α radiation
μ = 0.12 mm^−1^
*T* = 296 K
0.31 × 0.27 × 0.13 mm

#### Data collection

Bruker APEXII CCD diffractometer
Absorption correction: multi-scan (*SADABS*; Bruker, 2008 ▶) *T*_min_ = 0.962, *T*_max_ = 0.985
819 measured reflections
320 independent reflections
293 reflections with *I* > 2σ(*I*)
*R*_int_ = 0.022

#### Refinement

*R*[*F* ^2^ > 2σ(*F* ^2^)] = 0.037
*wR*(*F* ^2^) = 0.098
*S* = 1.09
320 reflections
37 parameters
H-atom parameters constrained
Δρ_max_ = 0.17 e Å^−3^
Δρ_min_ = −0.26 e Å^−3^

### 

Data collection: *APEX2* (Bruker, 2008 ▶); cell refinement: *SAINT* (Bruker, 2008 ▶); data reduction: *SAINT*; program(s) used to solve structure: *SHELXS97* (Sheldrick, 2008 ▶); program(s) used to refine structure: *SHELXL97* (Sheldrick, 2008 ▶); molecular graphics: *ORTEP-3* (Farrugia, 1997 ▶); software used to prepare material for publication: *SHELXL97*.

## Supplementary Material

Crystal structure: contains datablock(s) I, global. DOI: 10.1107/S1600536812008483/cv5246sup1.cif

Structure factors: contains datablock(s) I. DOI: 10.1107/S1600536812008483/cv5246Isup2.hkl

Supplementary material file. DOI: 10.1107/S1600536812008483/cv5246Isup3.cml

Additional supplementary materials:  crystallographic information; 3D view; checkCIF report

## Figures and Tables

**Table 1:** Hydrogen-bond geometry (Å, °)

*D*—H⋯*A*	*D*—H	H⋯*A*	*D*⋯*A*	*D*—H⋯*A*
N4—H4⋯N2^i^	0.86	2.10	2.943 (3)	166

Symmetry code: (i) 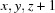.

**Fig. 1.**
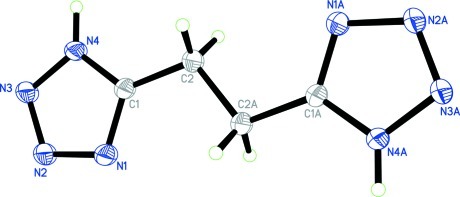
The molecular structure of I showing the atomic numbering and 30% probability displacement ellipsoids [symmetry code: (A) -x, y, -z].

## supplementary crystallographic information

### Comment

Due to the rich coordination modes and high content of nitrogen, tetrazole compounds have been studied for more than one hundred years and widely applied in coordination chemistry (Zhao *et al.*, 2008) and energetic materials (Singh *et al.*, 2006). As an important derivative of tetrazole, 5,5'-ethane-1,2-diylbis(1*H*-tetrazole) has been utilized to construct functional and interesting coordination compounds owing to its flexible coordination modes. The crystal structure of the title compound has been previously reported by Shen *et al.* (2011) in the monoclinic space group *P*2_1_/*c* (*Z* = 4). We report here the synthesis and crystal structure of a new polymorph of the title compound, (I).

Polymorph I crystallizes in the space group *C*2/*m* (*Z* = 2) with one-fourth part of the molecule in the asymmetric unit. In the crystal, all non-H atoms are situated on the mirror plane. Similar to the previously reported polymorph, each pair tetrazolate rings in I are coplanar linked by flexible —CH_2_—CH_2_— group (Fig. 1). The bond lengths and angles in I are within normal ranges and comparable with the previously reported structure. N—H···N hydrogen bonds (Table 1) link the adjacent molecules into one-dimensional belts along the *c* axis, whereas molecules of the previously reported polymorph are connected into a three dimensional supramolecular framework. The N···N distance [2.943 (3) Å] is longer than that seen in the previously published polymorph [2.840 (2) and 2.873 (2) Å] (Shen *et al.*, 2011). π-π stacking interactions between tetrazole rings of adjacent molecules [centroid-centroid distances = 3.4402 (10) Å] further consolidate the crystal packing.

### Experimental

The title compound was prepared from a mixture of sodium azide and succinonitrile using the method described by Chafin *et al.* (2008). Colourless block single crystals were obtained by recrystallization of the title compound from ethanol.

### Refinement

All H atoms were positioned geometrically (C—H = 0.97 Å and N—H = 0.86 Å) and refined as riding with *U*_iso_(H) = 1.2*U*_eq_(C,*N*).

### Crystal data

**Table:**

C_4_H_6_N_8_	*F*(000) = 172
*M**_r_* = 166.17	*D*_x_ = 1.644 Mg m^−^^3^
Monoclinic, *C*2/*m*	Mo *K*α radiation, λ = 0.71073 Å
Hall symbol: -C 2y	Cell parameters from 501 reflections
*a* = 10.951 (3) Å	θ = 3.7–25.9°
*b* = 6.678 (2) Å	µ = 0.12 mm^−^^1^
*c* = 5.0329 (14) Å	*T* = 296 K
β = 114.250 (4)°	Block, colorless
*V* = 335.58 (17) Å^3^	0.31 × 0.27 × 0.13 mm
*Z* = 2	

### Data collection

**Table:**

Bruker APEXII CCD diffractometer	320 independent reflections
Radiation source: fine-focus sealed tube	293 reflections with *I* > 2σ(*I*)
Graphite monochromator	*R*_int_ = 0.022
φ and ω scans	θ_max_ = 24.9°, θ_min_ = 3.7°
Absorption correction: multi-scan (*SADABS*; Bruker, 2008)	*h* = −7→12
*T*_min_ = 0.962, *T*_max_ = 0.985	*k* = −7→7
819 measured reflections	*l* = −5→5

### Refinement

**Table:**

Refinement on *F*^2^	Primary atom site location: structure-invariant direct methods
Least-squares matrix: full	Secondary atom site location: difference Fourier map
*R*[*F*^2^ > 2σ(*F*^2^)] = 0.037	Hydrogen site location: inferred from neighbouring sites
*wR*(*F*^2^) = 0.098	H-atom parameters constrained
*S* = 1.09	*w* = 1/[σ^2^(*F*_o_^2^) + (0.059*P*)^2^ + 0.15*P*] where *P* = (*F*_o_^2^ + 2*F*_c_^2^)/3
320 reflections	(Δ/σ)_max_ < 0.001
37 parameters	Δρ_max_ = 0.17 e Å^−^^3^
0 restraints	Δρ_min_ = −0.26 e Å^−^^3^

### Special details

**Table:**

Geometry. All e.s.d.'s (except the e.s.d. in the dihedral angle between two l.s. planes) are estimated using the full covariance matrix. The cell e.s.d.'s are taken into account individually in the estimation of e.s.d.'s in distances, angles and torsion angles; correlations between e.s.d.'s in cell parameters are only used when they are defined by crystal symmetry. An approximate (isotropic) treatment of cell e.s.d.'s is used for estimating e.s.d.'s involving l.s. planes.
Refinement. Refinement of *F*^2^ against ALL reflections. The weighted *R*-factor *wR* and goodness of fit *S* are based on *F*^2^, conventional *R*-factors *R* are based on *F*, with *F* set to zero for negative *F*^2^. The threshold expression of *F*^2^ > σ(*F*^2^) is used only for calculating *R*-factors(gt) *etc*. and is not relevant to the choice of reflections for refinement. *R*-factors based on *F*^2^ are statistically about twice as large as those based on *F*, and *R*- factors based on ALL data will be even larger.

### Fractional atomic coordinates and isotropic or equivalent isotropic displacement parameters (Å^2^)

**Table:**

	*x*	*y*	*z*	*U*_iso_*/*U*_eq_	Occ. (<1)
N1	0.19695 (18)	0.0000	−0.1482 (4)	0.0402 (6)	
N2	0.3293 (2)	0.0000	−0.0872 (4)	0.0407 (6)	
N3	0.4005 (2)	0.0000	0.1919 (4)	0.0393 (6)	
N4	0.31215 (19)	0.0000	0.3136 (4)	0.0355 (6)	
H4	0.3323	0.0000	0.4977	0.043*	
C1	0.1877 (2)	0.0000	0.1035 (5)	0.0327 (6)	
C2	0.0608 (2)	0.0000	0.1451 (5)	0.0378 (6)	
H2A	0.0582	0.1175	0.2559	0.045*	0.50
H2B	0.0582	−0.1175	0.2559	0.045*	0.50

### Atomic displacement parameters (Å^2^)

**Table:**

	*U*^11^	*U*^22^	*U*^33^	*U*^12^	*U*^13^	*U*^23^
N1	0.0237 (11)	0.0681 (14)	0.0269 (12)	0.000	0.0087 (9)	0.000
N2	0.0244 (11)	0.0676 (14)	0.0314 (12)	0.000	0.0129 (9)	0.000
N3	0.0238 (10)	0.0650 (13)	0.0305 (11)	0.000	0.0127 (8)	0.000
N4	0.0230 (10)	0.0589 (12)	0.0233 (10)	0.000	0.0082 (8)	0.000
C1	0.0227 (12)	0.0475 (13)	0.0256 (11)	0.000	0.0076 (9)	0.000
C2	0.0200 (13)	0.0643 (15)	0.0278 (13)	0.000	0.0085 (10)	0.000

### Geometric parameters (Å, º)

**Table:**

N1—C1	1.312 (3)	N4—H4	0.8600
N1—N2	1.353 (3)	C1—C2	1.488 (3)
N2—N3	1.297 (3)	C2—C2^i^	1.519 (4)
N3—N4	1.341 (3)	C2—H2A	0.9700
N4—C1	1.338 (3)	C2—H2B	0.9700
			
C1—N1—N2	106.33 (19)	N4—C1—C2	126.6 (2)
N3—N2—N1	110.93 (18)	C1—C2—C2^i^	111.4 (2)
N2—N3—N4	105.61 (18)	C1—C2—H2A	109.3
C1—N4—N3	109.32 (18)	C2^i^—C2—H2A	109.3
C1—N4—H4	125.3	C1—C2—H2B	109.3
N3—N4—H4	125.3	C2^i^—C2—H2B	109.3
N1—C1—N4	107.8 (2)	H2A—C2—H2B	108.0
N1—C1—C2	125.6 (2)		
			
C1—N1—N2—N3	0.0	N3—N4—C1—N1	0.0
N1—N2—N3—N4	0.0	N3—N4—C1—C2	180.0
N2—N3—N4—C1	0.0	N1—C1—C2—C2^i^	0.0
N2—N1—C1—N4	0.0	N4—C1—C2—C2^i^	180.0
N2—N1—C1—C2	180.0		

Symmetry code: (i) −*x*, −*y*, −*z*.

### Hydrogen-bond geometry (Å, º)

**Table:**

*D*—H···*A*	*D*—H	H···*A*	*D*···*A*	*D*—H···*A*
N4—H4···N2^ii^	0.86	2.10	2.943 (3)	166

Symmetry code: (ii) *x*, *y*, *z*+1.
